# Self-Perceived Nutritional Competency of Primary Healthcare Physicians in Qassim, Saudi Arabia

**DOI:** 10.7759/cureus.56145

**Published:** 2024-03-14

**Authors:** Abdulrhman Aloud, Chandra Sekhar

**Affiliations:** 1 Family and Community Medicine, Family Medicine Academy, Qassim Health Cluster, Buraidah, SAU

**Keywords:** saudi arabia, attitude of phc physicians, communication and counseling, skill, nutrition knowledge

## Abstract

Introduction: Overnutrition plays a vital role in the development of a spectrum of non-communicable diseases. Diet-related disorders have a huge impact on personal health as well as the country's economy for the management of such disorders. The study aims to assess the primary healthcare physicians' nutrition competency, which will be beneficial for evaluating the current situation and future strategies, training, nutrition care, and disease prevention.

Methodology: Among 147 primary healthcare physicians by simple random sampling from four cities and two rural areas of Qassim, Saudi Arabia, from December 2022 to December 2023 using a validated (NUTCOMP) tool. Data were entered, cleaned, and analyzed with SPSS software version 21.0 (IBM Corp., Armonk, NY). Informed consent was obtained from all study participants. Chi-square and ANOVA tests were applied to draw the significant differences.

Results: A total of 147 participants enrolled in this study, and the mean age and standard deviation (SD) of the study population were 34.38 ± 6.57. More than half of the physicians (n = 76, 51.7%) continued education on nutrition. Significant mean differences were observed between some and focused nutrition content received physicians versus no nutrition content received physicians concerning nutrition skill, communication, and nutrition attitude consecutively (P < 0.0001, P < 0.0001, and P < 0.0001). The mean nutrition knowledge, skill, communication, attitude score, and SD of PHCC physicians were 26.91 ± 5.42 (maximum 35), 31.19 ± 6.18 (maximum 40), 36.73 ± 7.48 (maximum 45), and 34.74 ± 6.23 (maximum 40), respectively.

Conclusions: Our study results show primary healthcare physicians perceive themselves to have good nutritional competency.

## Introduction

In the current era, the healthcare system demands that it provide safe, accessible, high-quality, and affordable care, especially when dealing with noncommunicable diseases (NCDs), which have huge clinical impacts and high financial burdens globally [1,2]. According to the World Health Organization (WHO), the probability of premature mortality from NCDs in Saudi Arabia (SA) reaches up to 21% [3].

Nutrition is an essential part of human development and well-being. Also, nutrition can be a threat to human health; diet-related disorders have a huge clinical impact on a wide variety of health conditions, ranging from dental caries to colorectal cancer (CRC). Thus, it is an important aspect of primary healthcare physicians, as their cornerstone role is in awareness and prevention [4-7].

Obesity is a global health threat that is highly linked to type 2 diabetes mellitus (T2DM), hypertension (HTN), dyslipidemia (DLP), depression, anxiety, sleep disorders, CRC, Alzheimer's disease, etc. In 2019, SA obesity and overweight prevalence were estimated to be 20.2% and 38.2%, respectively, which cost $3.8 billion, or 4.3%, of total health expenditures in SA in 2019 [8,9].

The number of diabetics in SA totals around 7 million [10] and prediabetics around 3 million, which costs about 14% of total government health spending annually and is expected to be higher in the future [11,12]. According to the WHO, 1.13 billion people worldwide have HTN, and in SA, HTN data were lacking. A national survey conducted in 2013 shows that around 15.2% of the population was hypertensive [13,14].

Although SA diet guidelines exist, most Saudis have unhealthy dietary habits, and the young population's consumption of refined food, meat, and sugar-sweetened beverages was high. Even more, dietary habits changed significantly during the COVID pandemic in SA; there was a 21% increase in consumption of carbohydrates and 13% higher fat consumption, increased intake of coffee and snacks, and less consumption of fruits and vegetables [15-17].

Nutrition care refers to advice or counseling regarding nutrition by healthcare professionals. Primary care or family physicians are expected to give nutritional advice, guidance, and counseling to be healthier, as the increasing presentation of diet-related chronic diseases is likely to increase the demand to provide nutrition care in the future. Also, some studies found patients prefer general practitioners more than dietitians to provide nutrition care [18,19].

Some studies found that brief physician nutrition counseling can produce beneficial outcomes on diet, weight, and blood lipids. Although physicians have limited time, lack of knowledge, lack of counseling skills, medical education, and patient noncompliance were the most identified barriers to providing nutrition care in primary health centers (PHCs) [20-23].

The higher consumption of fast and refined foods is usually correlated with higher economic status, perhaps due to a lack of nutritional awareness [24]. Hence, first-level contact physicians and their teams play a crucial role in nutrition care and health promotion. Competency is a combination of attitudes, communication, skills, and knowledge that determines a clinician’s ability to perform efficient and safe interventions [25].

Given the above situation, we aim to study the nutritional competency of primary healthcare physicians in the Qassim region to provide the best health promotional measures in addition to regular practice at PHCs.

## Materials and methods

Study design, setting, and sampling

This was a cross-sectional study conducted in a primary healthcare center in Al Qassim province, Saudi Arabia. The primary healthcare centers (PHCC) were selected from four major cities (Buraydah, Unaizah, Alrass, and Albadya). These four cities serve to represent the maximum Qassim population and also to represent the comprehensive picture of the province, including the rural distribution of participants from rural areas (Albukharya, Alhilaliah). Centers were selected through a simple random sampling technique. Physicians who were on duty in the selected centers during the data collection visit were invited to participate and fill out the study tool using online Google Forms. Furthermore, family medicine residents at Qassim Family Medicine Academy were approached through WhatsApp and invited to participate.

The inclusion criteria were primary healthcare physicians who belonged to selected cities and rural areas of Qassim province and were willing to participate. Physicians who were not willing to participate and interns were not included.

Sample size

The sample size was estimated according to the Ministry of Health Statistics. Primary healthcare has 155 centers working in Qassim province, and those centers accommodate approximately 568 primary care doctors, which is inclusive of 60 family medicine residents. Out of these numbers, 25% of physicians were included in the study. According to 25% (n = 142), the study population size was 568/25 = 142 doctors. However, 147 samples were completed to maintain the accuracy of the study.

Data collection tool

A validated questionnaire (NUTCOMP tool) was adopted from the previous study from Australia, specially designed to assess self-perceived nutrition competence [25]. Permission was obtained to use the NUTCOMP tool.

Demographic data (age, gender, nationality, and professional qualification) and the status of nutrition education and the provision of nutrition care were included in the first section of the questionnaire. The second section of the questionnaire included seven nutritional knowledge questions. The third section had eight nutritional skill questions. In the fourth section, there are nine nutritional communication and counseling questions. In the fifth section, there are eight nutrition attitude questions.

The responses for the last four sections used a 5-point Likert scale. Means and standard deviation were calculated based on scores ranging from 1 to 5 (1 = not confident, 2 = not very confident, 3 = somewhat confident, 4 = very confident, and 5 = extremely confident). 

Pilot study

After obtaining the facilitation letter, a pilot study was conducted among 10 physicians for technical feasibility, ensuring a good presentation and order of the questions. The pilot study sample was not included in the main study sample.

Ethical considerations

Before data collection for the study, ethical committee approval was guaranteed by the Qassim Regional Ethics Committee, with approval number 607-44-8011. Informed consent was obtained from all participants.

Statistical analysis

Data were entered and analyzed using SPSS software 21.0 version (IBM Corp., Armonk, NY). Means and standard deviations were calculated for the continuous variables of nutritional knowledge, skill, communication, and attitude in the study. Proportion and frequency were used to present categorical data. A chi-square test was applied to assess the relationship among the categorical variables. The level of statistical significance was set at a P-value of less than 0.05 with a 95% confidence interval.

## Results

In the present study, 147 primary healthcare physicians participated. The response rate in the study population was 81.6% (147/180). Cronbach’s alpha test for the internal consistency of seven knowledge questions had a value of 0.847; for eight nutrition skills questions, the value was 0.845. Similarly, for the nine questions of nutrition communication and counseling, the value was 0.919, and lastly, for the eight nutrition attitude questions, Cronbach's alpha value was 0.912. The mean age and standard deviation of the study population were 34.38 ± 6.57. The male physician distribution in the study was 53.1% (n = 78). More than half were general practitioners, 55.1% (n = 81) (Table 1).

**Table 1:** Demographic characteristics among the study population.

Variables	Number	Percentage
Age mean ± SD	34.38 ± 6.57	
Gender: male	78	53.1
Female	69	46.9
Nationality: Saudi	81	55.1
Non-Saudi	66	44.9
Position: general practitioner	81	55.1
FM resident	42	28.6
FM specialist	15	10.2
FM consultant	9	6.1

Table 2 depicts that nearly 20.4% (n = 30) of study participants completed a medical college program that did not include nutrition content. About 51.7% (n = 76) of the study population participated in continuing education on the topic of nutrition. For the question of further nutrition education to support my current role, the mean and standard deviation of the responses were 3.51 ± 1.31.

**Table 2:** Status of previous nutrition education and its domains in the study population.

Previous nutrition education	Yes (%)	No (%)
Completed program that did not include nutrition content	30 (20.4)	117 (79.6%)
Completed program that included some nutrition content	106 (72.1)	41 (27.9%)
Completed a program that was predominantly focused on nutrition	11 (7.5)	136 (92.5%)
Have you ever participated in continuing education on the topic of nutrition?	76 (51.7)	71 (48.3%)
Response	Number	Percentage
I need further nutrition education to support my current role		
Strongly disagree	18	12.2
Disagree	12	8.2
Neither agree nor disagree	34	23.1
Agree	42	28.6
Strongly agree	41	27.9
Mean ± SD	3.51 ± 1.31	
Over a given month, provision of nutrition care to the patients		
Never	3	2.0
Rarely	23	15.6
Half the time	52	35.4
Often	38	25.9
Most of time	31	21.1
Time spent on nutrition care for each patient during the visit		
<1 minute	28	19.0
1–3 minutes	74	50.3
3–5 minutes	40	27.2
5–10 minutes	4	2.7
>10 minutes	1	.7

Table 3 stated that two-thirds of physicians (n = 98, 66.6%) were confident in using guidelines for nutrition-related specific chronic diseases, and a smaller proportion of physicians (n = 60, 40.8%) were confident about recently published peer-reviewed evidence regarding nutrition and chronic diseases.

**Table 3:** Nutritional competency knowledge among the study population.

Knowledge variables	Not confident at	Not very confident	Some what confident	Very confident	Extremely confident	Mean ± SD
How are different body systems affected by foods and nutrients?	1 (0.7)	6 (4.1)	32 (21.7)	37 (25.2)	71 (48.3)	4.16±0.95
How do foods and nutrients influence the development and management of chronic diseases?	1 (0.7)	4 (2.7)	13 (8.8)	42 (28.6)	87 (59.2)	4.42±0.81
How can an individual’s body composition (including size, shape, and weight) impact the development of chronic disease?	2 (1.4)	6 (4.1)	15(10.2)	34 (23.1)	90 (61.2)	4.38±0.92
The Dietary Guidelines for Saudis, including number of recommended serves of food groups and serving sizes for different ages and genders	13 (8.8)	27 (18.4)	52 (35.4)	28 (19)	27 (18.4)	3.19±1.19
Guidelines for the nutrition-related management of specific chronic diseases (including type 2 diabetes and cardiovascular disease)	4 (2.7)	10 (6.8)	35 (23.8)	49 (33.3)	49 (33.3)	3.87±1.03
How foods and nutrients interact with medications?	3 (2.0)	21 (40.3)	35 (23.8)	43 (29.3)	45 (30.6)	3.72±1.10
The most recently published peer-reviewed evidence regarding nutrition and chronic disease	23 (15.6)	26 (17.7)	38 (25.9)	27 (18.4)	33 (22.4)	3.14±1.36

Table 4 revealed that 61.2% (n = 90) of physicians who collected diet histories or used food frequency questionnaires were confident and above, and approximately 66.7% (n = 98) of physicians recommended changes in the food choices of patients with chronic disease as confident and above. 

**Table 4:** Opinions of primary healthcare center physicians on nutritional skill among the study group.

Skill variables	Not confident at	Not very confident	Somewhat confident	Very confident	Extremely confident	Mean ± SD
Interpret data about height, weight, and body composition against reference ranges	2 (1.4)	9 (6.1)	12 (8.2)	27 (18.4)	97 (66)	4.41±0.97
Interpret an individual’s biological data (e.g., blood pressure, cholesterol levels) against reference ranges	1 (0.7)	4 (2.7)	10 (6.8)	21 (14.3)	111 (75.5)	4.61±0.78
Collect information on the food that an individual usually eats (e.g., diet history, food frequency questionnaire)	8 (5.4)	18 (12.2)	31 (21.1)	20 (13.6)	70(47.6)	3.85±1.28
Use the Dietary Guidelines for Saudis to evaluate the appropriateness of an individual’s food intake	17 (11.6)	38 (25.9)	43 (29.3)	22 (15)	27 (18.4)	3.02±1.27
Determine appropriate food or nutrition goals for an individual with chronic disease	3 (2)	5(3.4)	38 (25.9)	42 (28.6)	59(40.1)	4.01±0.99
Formulate a meal plan for an individual with chronic disease	7 (4.8)	16 (10.9)	37 (25.2)	31 (21.1)	56 (38.1)	3.76±1.20
Recommend changes in food choices for an individual with chronic disease	5 (3.4)	11 (7.5)	33 (22.4)	36 (24.5)	62(42.2)	3.94±1.12
Monitor and evaluate changes over time regarding the food an individual usually eats	7 (4.8)	23 (15.6)	42 (28.6)	32 (21.8)	43 (29.3)	3.55±1.20

Table 5 showed that about 73.5% (n = 108) physicians were confident and above in checking patient’s understanding of the influence of food on their health; nearly 76.9% (n = 113) physicians were confident and above in identifying individuals who need additional support from other health professionals.

**Table 5:** Status of nutrition communication and counselling in the study population.

Communication variables	Not confident at	Not very confident	Somewhat confident	Very confident	Extremely confident	Mean ± SD
Clearly describe what patients can expect from their discussions with you about food or nutrition	1 (0.7)	8 (5.4)	30 (20.4)	42(28.6)	66(44.9)	4.11±0.96
Check a patient’s understanding of the influence of food and nutrients on their health	5 (3.4)	11 (7.5)	23 (15.6)	37 (25.2)	71 (48.3)	4.07±1.11
Work with patients to identify possible ways to improve the food they usually eat	6 (4.1)	8(5.4)	28(19)	45(30.6)	60(40.8)	3.98±1.09
Demonstrate genuine empathy to patients about their food-related experiences and goals	6(4.1)	13(8.8)	22(15)	44(29.9)	62(42.2)	3.97±1.14
Maintain a non-judgmental attitude in discussions with patients about the food they eat	2(1.4)	7(4.8)	27(18.4)	35(23.8)	76(51.7)	4.19±0.99
Communicate with patients about food and nutrition using culturally appropriate language	2(1.4)	5(3.4)	25(17)	39(26.5)	76(51.7)	4.23±0.94
Consider how personal, social, cultural, psychological and economic factors may influence the foods that a patient eats	7(4.8)	7(4.8)	32(21.8)	38(25.9)	63(42.9)	3.97±1.12
Identify individuals who need additional support from other health professionals or services regarding the food they eat	3(2.0)	7(4.8)	24(16.3)	40(27.2)	73(49.7)	4.17±1.00
Communicate with other health professionals about the discussions you’ve had with patients regarding food	6(4.1)	14(9.5)	28(19)	25(17)	74(50.3)	4.0±1.20

Table 6 depicts that the majority of physicians, 82.3% (n = 121), agreed or above that it is important to eat healthy foods regardless of age, body weight, and physical activity level. Most physicians, 77.6% (n = 114), agreed that encouraging patients to eat healthy foods is an effective use of their professional time.

**Table 6:** Status of primary healthcare center physicians attitude towards care in the study population.

Attitude variables	Completely disagree	Somewhat disagree	Neither agree or nor disagree	Somewhat agree	Completely agree	Mean ± SD
It is important that all individuals usually eat healthy foods regardless of age, body weight, and physical activity levels	2(1.4)	12(8.2)	12(8.2)	24(16.3)	97(66)	4.37±1.02
If the topic arises, it is important that I encourage my patients to eat healthy foods	0(0)	4(2.7)	21(14.3)	20(13.6)	102(69.4)	4.49±0.83
It is important that I take every opportunity possible to encourage my patients to eat healthy foods	6(4.1)	3(2)	18(12.2)	31(21.1)	89(60.5)	4.32±1.04
Encouraging my patients to eat healthy foods is an effective use of my professional time	6(4.1)	4(2.7)	23(15.6)	31(21.1)	83(56.5)	4.23±1.07
Providing specific nutrition recommendations to my patients that can assist with managing their chronic disease is an effective use of my professional time	4(2.7)	5(3.4)	21(14.3)	32(21.8)	85(57.8)	4.28±1.01
Encouraging my patients to eat healthy foods is within my scope of practice	2(1.4)	8(5.4)	24(16.3)	38(25.9)	75(51)	4.19±0.99
Providing specific nutrition recommendations to my patients that can assist with managing their chronic disease is within my scope of practice	5(3.4)	3(2)	17(11.6)	34(23.1)	88(59.9)	4.34±0.99
It is important that I encourage my patients to seek support from other health professionals if I am unable to meet their nutrition-related needs	2(1.4)	5(3.4)	16(10.9)	19(12.9)	105(71.4)	4.49±0.91

Table 7 stated that of physicians who spent <3 minutes, 41.2% (n = 42) were providing nutrition care half of the time; of those physicians who spent >3 minutes, 40% (n = 18) were providing nutrition care most of the time. There was a statistically significant association observed between time spent on nutrition and providing nutrition care to the physicians (P<0.05).

**Table 7:** Association between time spent on nutrition versus providing nutrition care in the study population. X^2^ = 20.07, 4df, P < 0.0001.

Time spent on nutrition	Never	Rarely	Half the time	Often	Most of time
<3 minutes	2 (2%)	21 (20.6%)	42 (41.2%)	24 (23.5%)	13 (12.7%)
>3 minutes	1 (2.2%)	2 (4.4%)	10 (22.2%)	14 (31.1%)	18 (40%)
Total	3 (2%)	23 (15.6%)	52 (35.4%)	38 (25.9%)	31 (21.1%)

Table 8 stated that the mean knowledge score and SD among the physicians who received no nutrition content were 22.93 ± 6.11, whereas for physicians who focused on nutrition, the mean and SD of the knowledge score were 30.36 ± 4.84, and this association was statistically significant (P<0.05). Statistically significant mean differences were observed in physician's nutrition knowledge, skill, communication, and nutrition attitude between physicians who completed a medical program with no nutrition content versus some nutrition content and focused nutrition content consecutively (P < 0.0001, P < 0.0001, P < 0.0001, and P < 0.0001).

**Table 8:** Status or previous nutrition education about mean nutrition knowledge, skill, communication, and nutrition attitude in the study population.

Previous nutrition	Sample (N)	Mean	SD	P-value	Confidence interval
No nutritional content	30	22.93	6.11	0.0001	20.64–25.21
Some nutrition content	106	27.68	4.67		26.78–28.58
Focused on nutrition	11	30.36	4.84		27.11–33.61
Total	147	26.91	5.42		26.0–27.80
No nutritional content	30	27.06	5.99	0.0001	24.82–29.30
Some nutrition content	106	32.07	5.79		30.96–33.19
Focused on nutrition	11	33.90	5.94		29.91–37.90
Total	147	31.19	6.18		30.18 - 32.19
No nutrition content	30	31.80	9.46	0.0001	28.26–35.33
Some nutrition content	106	37.89	6.33		36.67–39.11
Focused on nutrition	11	39.00	6.58		34.57–43.42
Total	147	36.73	7.48		35.51–37.95
No nutritional content	30	30.70	8.34	0.0001	27.58–33.81
Some nutrition content	106	35.83	4.94		34.88–36.79
Focused on nutrition	11	35.18	6.83		30.58–39.77
Total	147	34.74	6.23		33.72–35.75

## Discussion

Nutritional care is an important aspect of healthcare, especially for non-communicable diseases. Integration of physician knowledge, skill, communication, and attitude plays a very important role in preventive and therapeutic intervention. This study fulfilled its aim to assess nutritional competency among primary healthcare physicians during the period from December 2022 to December 2023.

In our study, approximately half of the study population, 51.7% (n = 76), continued education on nutrition. A study conducted in Jeddah in 2019 stated that 35.6% continued medical education on nutrition, and a smaller percentage could be due to the smaller sample size and the study conducted four years ago [26]. Another multicentric study among eight developed countries was published in 2019 and revealed that only 39% of participants received training on nutrition [21].

In the current study, nearly one-fifth of the participants, or 20.4%, completed a medical curriculum that did not include nutrition content. In Jeddah, a study showed that 16.7% did not include nutrition content in their medical curriculum [26]. A study conducted at GCC also mentioned that 36% of physicians opined that the medical curriculum is a primary source of nutrition knowledge and recommended CME activities for family physicians on nutrition topics [27].

In our current study, two-thirds (66.6%) of physicians were confident in using guidelines for nutrition-related, specific chronic diseases. Also, the mean knowledge score of nutrition among the PHCC physicians was 26.91 ± 5.41, out of a maximum score of 35. A study conducted by Al-Gassimi et al. [26] revealed that the mean nutrition knowledge score was observed to be 25.8 ± 5.4. A low nutrition knowledge score of 35.8% was identified in a study conducted in Croatia [28]. Similar studies were conducted in GCC countries like Kuwait [29], Qatar [30], and Saudi Arabia [31], wherein, respectively, 60%, 64%, and 52.1% of participants were queried regarding nutrition knowledge. Less scores in Al-Zahrani's earlier published study in 2009 [31]. In our study, a higher nutrition knowledge score of 71.4% was observed. 

A smaller proportion of physicians, 40.8% (n = 60), were confident about the question of recently published peer-reviewed evidence regarding nutrition and chronic diseases in our study. In the same context as the finding, some systematic reviews were conducted on educational interventions for the improvement of nutrition care in the United States of America (USA) in 2016 [32], another study in the same dimension from the United Kingdom (UK) published in 2020 [33], and another study from physicians from New Zealand and Australia [34] highlighted that PHCC physicians require nutrition education and access to evidence-based information. 

In the nutritional skill domain, 61.2% (n = 90) of physicians who collected diet histories or used food frequency questionnaires were confident or above. Ninety-eight (66.7%) physicians recommend changes in the food choices of patients with chronic disease as confident and above. Systematic review studies conducted in the UK also stated that very few physicians were confident in using dietary guidelines [33]. In 2012, an Australian study emphasized the need for nutritional competencies to be introduced in medical courses to strengthen the nutritional skills of physicians [35].

Concerning the nutritional counseling domain, 76.9% (n = 113) of physicians were confident and above in identifying individuals who need additional support from other health professionals. A study conducted in Australia by Ball et al. stated that general practitioners are preferred more than dietitians to provide nutrition care [18]. Ockene et al. found that physician nutrition counseling can improve diet, weight, and blood lipid outcomes in individuals [20]. Some studies conducted in different parts of the world confirmed that physicians' nutrition knowledge, counseling skills, and education are crucial for health [21-23]. The nutrition counseling domain among FM residents was higher than among internal medicine (IM) and obstetrics and gynecology (OB-gyn) residents at Cleveland University, USA [36].

About three-fourths of the sample of participating physicians agreed that encouraging patients to eat healthy foods is within their scope of practice. Another Australian study stated that strategies for improving doctor collaboration and nutritional interventions with dietitians will enhance health outcomes [37]. Also, a similar observation in AlGassimi's study denoted the gap between capabilities and the actual practice of nutrition care [26]. Most of the studies perceived that attitude plays a vital role in achieving nutritional competencies.

Regarding strengths, a validated questionnaire was used, which included multiple cities and rural areas within the context of Qassim. Among its limitations, a self-administered questionnaire has a chance of misunderstanding some questions. Further studies are required to substantiate results regarding the generalisability of study findings. Our results give further direction on PHC-level implementation of preventive goals.

## Conclusions

Our study investigates the nutrition competency of primary healthcare physicians, recognizing the significant role of nutrition in non-communicable diseases. Conducted through a cross-sectional approach from December 2022 to December 2023, the findings revealed that over half of the physicians continued education on nutrition, significantly impacting their nutrition knowledge, skill, communication, and attitude. The study concludes that primary healthcare physicians have good nutritional competency. However, it emphasizes the need for further research to validate these self-perceived competencies, contributing to the existing literature on the importance of ongoing nutrition education for healthcare professionals in the context of preventing and managing diet-related disorders.
